# Different outcome of sarcoglycan missense mutation between human and mouse

**DOI:** 10.1371/journal.pone.0191274

**Published:** 2018-01-23

**Authors:** Sara F. Henriques, Cécile Patissier, Nathalie Bourg, Chiara Fecchio, Doriana Sandona, Justine Marsolier, Isabelle Richard

**Affiliations:** 1 INTEGRARE, Genethon, Inserm, Univ Evry, Université Paris-Saclay, Evry, France; 2 Department of Biomedical Sciences, University of Padova, Via U. Bassi, Padova, Italy; University of Minnesota Twin Cities, UNITED STATES

## Abstract

Sarcoglycanopathies are rare autosomic limb girdle muscular dystrophies caused by mutations in one of the genes coding for sarcoglycan (α, β, δ, and γ-sarcoglycans). Sarcoglycans form a complex, which is an important part of the dystrophin-associated glycoprotein complex that protects sarcolemma against muscle contraction-induced damages. Absence of one of the sarcoglycan at the plasma membrane induces the disappearance of the whole complex and perturbs muscle fiber membrane integrity. We previously demonstrated that point mutations in the human sarcoglycan genes affects the folding of the corresponding protein, which is then retained in the endoplasmic reticulum by the protein quality control and prematurely degraded by the proteasome. Interestingly, modulation of the quality control using pharmacological compounds allowed the rescue of the membrane localization of the mutated sarcoglycan. Two previously generated mouse models, knock-in for the most common sarcoglycan mutant, R77C α-sarcoglycan, failed in reproducing the dystrophic phenotype observed in human patients. Based on these results and the need to test therapies for these fatal diseases, we decided to generate a new knock-in mouse model carrying the missense mutation T151R in the β-sarcoglycan gene since this is the second sarcoglycan protein with the most frequently reported missense mutations. Muscle analysis, performed at the age of 4 and 9-months, showed the presence of the mutated β-sarcoglycan protein and of the other components of the dystrophin-associated glycoprotein complex at the muscle membrane. In addition, these mice did not develop a dystrophic phenotype, even at a late stage or in condition of stress-inducing exercise. We can speculate that the absence of phenotype in mouse may be due to a higher tolerance of the endoplasmic reticulum quality control for amino-acid changes in mice compared to human.

## Introduction

In eukaryotic cells, proteins acquire their 3D structure through several steps of folding of the polypeptide chain thanks in particular to the help of chaperone proteins. The folding process is under the surveillance of several quality control systems depending on the intracellular location of the nascent protein. Single amino acid substitutions can affect this folding process leading to protein misfolding. Depending on the novel acquired properties of the mutated protein, the folding defective polypeptide either can accumulate into the cell, or be prematurely eliminated by degradative pathways. Thus, missense mutations in a large number of proteins have been described to result in protein-misfolding diseases [1]. In particular, this is the case for the sarcoglycanopathies, a group of recessive limb-girdle muscular dystrophies (LGMD2- D, E, C and F) caused by genetic defects in the genes coding for α, β, γ and δ-sarcoglycans (SG), respectively [2, 3]. These proteins form hetero-tetrameric complexes in skeletal and cardiac muscles [4, 5], and are part of the dystrophin-associated glycoprotein complex (DGC). The DGC participates in the link between the cytoskeleton and the extracellular matrix, which is essential for the integrity of muscle membrane during contraction [6, 7]. Mutations in one of the four sarcoglycans destabilize the complex and weaken the capacity of membrane to withstand mechanical stress [8–10], leading to death of the muscle fibers and subsequent loss of muscle mass. No treatments are currently available to cure sarcoglycanopathies.

One of the most frequently reported mutations in sarcoglycanopathies causes the substitution of an arginine by a cysteine in position 77 (R77C) in α-SG. It has been demonstrated that the R77C mutant α-SG, being misfolded, is recognized by the Endoplasmic Reticulum Quality Control (ERQC) system in human, trapped in the ER [11] and subsequently degraded by the Endoplasmic Reticulum Associated Degradation (ERAD) system [12, 13]. We showed that it is possible to pharmacologically rescue from early degradation this particular α-SG mutant as well as other SG mutants, leading to restoration of its membrane localization [12, 14]. We and others have previously generated mouse knock-in (KI) models corresponding to this mutation by replacement of the amino-acid at position 77 of the α-SG by a cysteine [12, 15]. Unexpectedly, correct localization at the membrane of the mutant SG protein and of the complex and absence of a dystrophic phenotype were observed in both mouse models. This lack of phenotype is not totally understood, especially since the sarcoglycan function is not dispensable in mice as indicated by the severe phenotype of the *Sgca* knock-out (KO) model [16].

**Fig 1:**
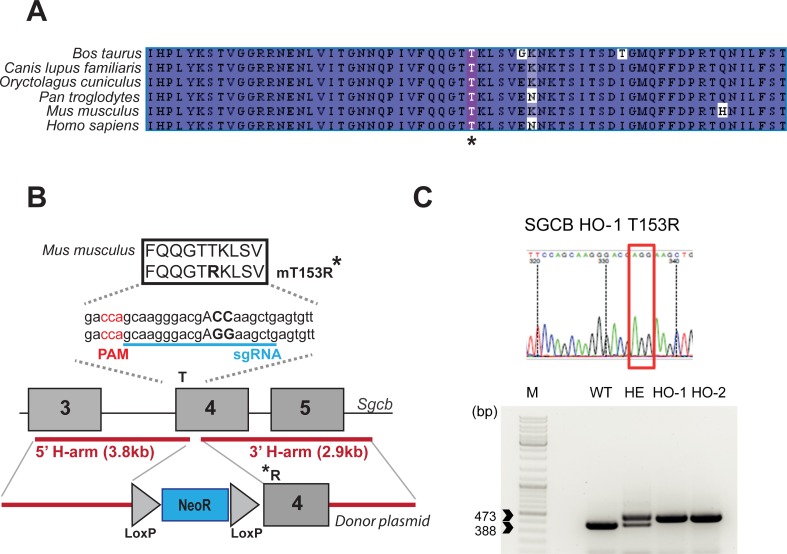
Generation of the T153R β-SG KI mouse model. (**A**) Clustal-Omega alignment between β-SG protein sequences of different vertebrate species shows a high degree of conservation of the β-SG protein at the vicinity of the human T151R mutation indicated by an asterisk. The higher to lower degree of homology is indicated by the dark to light blue color. (**B**) Strategy for the generation of the T153R β-SG KI mouse model. Briefly, homologous recombination event was induced using CRISPR/Cas9 based-cleavage at the mutation site and a homologous donor plasmid bearing the desired mutation and a self-excision LoxP-flanked Neomycin resistant cassette. (**C**) Sequencing and PCR analysis shows the correct ACC to AGG substitution as well as the residual LoxP sites.

To define whether the absence of abnormality with the first KI model was specific to *Sgca* or to the particular mutation, we decided to develop a new sarcoglycan KI mouse model. We selected the missense mutation T151R in the β-SG gene (*SGCB*) since β-SG is the second sarcoglycan protein with the most frequently reported missense mutations. This mutation induces a severe phenotype in patients and is located in a highly conserved region (**Fig 1A**). The corresponding mouse model was created by the CRISPR/Cas9 technology. Muscle analysis at the age of 4 and 9-months revealed the correct presence at the muscle membrane of the mutant β-SG protein and of the other components of the DGC complex. In accordance with the presence of the SG complex at the membrane and similarly to the previous *Sgca* KI mouse model, these mice do not develop a LGMD-like phenotype. These observations suggest that the maturation of the sarcoglycan proteins present a species-to-species difference, with a higher tolerance for amino-acid changes in mice.

## Materials and methods

### Generation of a new mouse model for sarcoglycanopathies

To generate a T153R β-SG mouse model, a self-excision lox-neomycin cassette carrying the desired mutation (i.e., ACC codon to AGG change at position 458/459) was introduced in exon 4 of the *Sgcb* gene (**Fig 1B**). Because of the presence of repeated regions at the locus, the cassette carries relatively short arms of homology (3.8 and 2.9 kb). Therefore, to increase the homologous recombination efficiency, we designed a guide RNA (sgRNA) covering the area of the mutation: (5’-**CCA**GCAAGGGACGACCAAGCTGA-3’; the PAM sequence is indicated in bold and the position of the targeted codon underlined) which was cloned in a plasmid carrying the *Streptococcus pyogenes* Cas9 (SpCas9) coding sequence. The self-excision lox-neomycin cassette was introduced in ES cells by electroporation together with the sgRNA-SpCas9 plasmid. After screening by PCR, southern blot and sequencing, three clones were validated. The clone 1C7 was injected in blastocysts that were then implanted in foster mice. The resulting chimeric males were crossed with C57BL6/N to obtain the heterozygous animals that were then interbreed (B6;129-*Sgcb*^Tm1GNT^). For genotyping, a specific set of primers was used to amplify genomic tail DNAs extracted from littermates of WT, heterozygous and homozygous genotypes: 1326_ScM_01_Fwd: 5’-AGCCGTGTTCCCTGTGACCTGT-3’ and 1326_ScM_01_Rev: 5’-GCGTCTCATAGTCCGTGCTGA-3’. All mice were handled according to the European guidelines for the human care and use of experimental animals, and all procedures on animals were approved by the French ethics committee n°51 and by the French Ministry of “Education Nationale, Enseignement et Recherche” under the number APAFIS#3491–2016010815087043. Animals were housed in a barrier facility with 14-h light, 10-h dark cycles, and provided food and water *ad libitum*.

### Eccentric exercise experiment

Groups of WT and KI male and female mice (n = 5 per condition) were subjected to an eccentric exercise on a treadmill 3 consecutive days in the following conditions, 15° downhill at a speed of 10m/min for 30 min. Just after the last bout of exercise, Evans Blue was injected by intraperitoneal injection (1mg/g of mice). Mice were sacrificed the day after and muscles were sampled.

### RNA and DNA extraction and qPCR

Total RNA was prepared using 1 ml of Trizol reagent (Invitrogen). The RNA was extracted following the manufacture’s protocol. One μg total RNA was used as template for reverse transcription using the RevertAid First Strand cDNA Synthesis Kit (Life technologies). Following reverse transcription, 1 μl of each cDNA was used as template for qPCR using primers and Taqman probes to α-SG (Mm00486068_m1 Sgca), β-SG (Mm00449389_m1 Sgcb), γ-SG (Mm00488741_m1 Sgcg) or TaqMan gene expression assays: Mm00481256_m1, Mm00438095_m1, Mm01341361_m1, Mm00434228_m1, Mm00711678_m1, Mm01329494_m1 and Mm00434455_m1 for TMEMC8, CD3g, TIMP1, IL1b, COL6A3, Myh8 and CD11b genes, respectively. PCR was carried out using the ABsoluteQPCR ROXMix (Thermo Scientific). The ubiquitous acidic ribosomal phosphoprotein (P0, Forward 5’-CTCCAAGCAGATGCAGCAGA-3’; Reverse 5’-ATAGCCTTGCGCATCATGGT-3’; Probe 5’-CCGTGGTGCTGATGGGCAAGAA-3’) was amplified to normalize the results for mRNA. All PCR reactions were performed in duplicate and each quantification repeated three times.

### Immunoblot analysis

Total proteins were extracted with RIPA lysis buffer (89900, Thermo Scientific) supplemented with 1X Protease Inhibitor—Complete ULTRA tablets mini (5892791001, Roche) and 1X Benzonase nuclease HC (712063, Millipore) for 1 h at 4°C. Equal amounts of total cellular proteins were resolved on NuPAGE® Novex® 4–12% Bis-Tris Protein Gels (NP0336BOX, Thermo Fisher Scientific) and transferred to nitrocellulose membranes (iBlot, Thermo Fisher Scientific) following the manufacturer’s instructions. Membranes were then blocked in Odyssey Blocking Buffer (927-4-0000, Li-Cor) for 1 h at room temperature. Incubation with primary antibodies was carried out at 4°C overnight in Odyssey Blocking Buffer. The following antibodies were used: rabbit anti-β-SG (dilution 1/100, HPA011422, Sigma) and rabbit anti-Actinin alpha (H-300) (dilution 1/1000, sc-15335, Santa Cruz). After 1 h incubation with donkey anti-rabbit 680 antibody (926–68073, EuroBio) at room temperature, proteins were detected by fluorescence (Odyssey, Li-Cor) following the manufacturer’s instructions. Western blot signal quantification was performed with the Plot Lanes Analysis tool of Image J software (NIH).

### Histology and immunostaining

Skeletal muscles were dissected out and frozen in isopentane cooled in liquid nitrogen. Transverse cryosections (8 or 10 μm thickness) were prepared from frozen muscles. Mouse muscle sections were rehydrated with PBS for 5min at room temperature. The endogenous peroxidases were inhibited by H_2_O_2_ (S 2023, DAKO®) for 20 min at room temperature and rinse with PBS for 5 min. Slides were then blocked for 15 min with PBS with mouse IgG blocking agent (MOM MKB-2213, VECTOR) to prevent non-specific staining and then rinsed with PBS. These slides were incubated overnight at 4°C with mouse anti-α-SG (1/40, NCL-a-sarc, Novocastra), anti-β-SG (1/40, NCL-L-BSARC, Novocastra) or anti-Dystrophin (1/40, NCL-DYS2, Novocastra) in PBS. After washing three times in PBS, the slides were incubated with EnVision™ Mouse HRP (K 4000, DAKO) for 30 min at room temperature and in the dark. Slides were subsequently washed three times in PBS. The slides were incubated for 2 to 5 min at room temperature with DAB reagent (K 3466, DAKO) diluted in the appropriate solution following manufactures’ protocol, and mounted on slides with Eukitt® mounting liquid (03989, Sigma). Images were acquired with a Zeiss Axio Scan.Z1, Slide Scanner. Cryosections were also processed for Hematoxylin-Phloxine-Saffron (HPS), Sirius Red or for detection of Evans Blue positive fibers. Digital images of the entire sections were captured with an Axioscan microscope (Leica). To minimize subjective bias, all analyses were performed independently by 2 different persons.

### Protein alignment

The dataset is composed of the complete protein sequences of 6 organisms downloaded from NCBI website: *Homo sapiens* (CAG33091.1), *Mus musculus* (EDL37860.1), *Pan troglodytes* (XP_517299.2), *Oryctolagus cuniculus* (NP_001075825.1), *Canis lupus familiaris* (XP_853790.1) and *Bos taurus* (NP_001095658.1). Sequences were aligned with Clustal Omega (version 1.2.4) [17] and the alignment was formatted using Jalview (version 2.10.1) [18].

### Data and statistical analysis

The GraphPad PRISM 7.01 program (GraphPad Software Inc. La Jolla, CA, USA) was used for statistics. The results presented in all the figures represent the average ± SEM of at least three independent experiments. One-way ANOVA test for multiple comparisons tests and the p-values were calculated using the approach of Dunnett for multiple comparisons with the WT as control column (p <0.05).

## Results

Because of the lack of phenotype in the H77C α-SG mouse model and with respect to the need of an adequate model for evaluating pharmacological strategies able to rescue missense mutations leading to sarcoglycanopathies, we decided to create a new KI model that recapitulates a human missense mutation (T151R, Threonine to Arginine at position 151) in the β-SG protein. The choice of the sarcoglycan and of the mutation was based on several elements. First, β-SG is the second sarcoglycan protein with the most frequently reported missense mutations. Therefore, evidence of therapeutic efficiency for rescue of missense mutations gathered for this protein will have an impact for more patients that for γ–sarcoglycan for which most of the mutations induce a frameshift or for δ-sarcoglycan for which very few patients have been reported so far [19]. Second, T151R β-SG mutant induces a severe phenotype in patients often associated with cardiomyopathy and sometimes associated with respiratory insufficiency [20], indicating a high impact of the mutation on the protein and thus theoretically increasing the chance to obtain a phenotype in mouse. Third, the residue is located in a highly conserved region between human and mouse as well as in other species (**Fig 1A**), again theoretically increasing the chance to obtain a similar impact on the murine protein than in the human one. Of note, the β-SG protein presents a 95% identity between mouse and human whereas the identity is 89% for α-SG. Finally, we previously showed that this mutant is pharmacological rescuable in human cell lines [14]. Note that, in mice, the corresponding mutation is located not at position 151 but 153 since the murine cDNA carries two supplementary amino-acids at the N-terminus of the protein.

We introduced a T to R modification at position 153 in β-SG protein by changing the ACC codon to AGG at position 458/459 in the exon 4 of the *Sgcb* gene through a strategy combining homologous recombination and targeting of the locus using the CRISPR/Cas9 system (**Fig 1B**). Mutant mice were viable and obtained at normal ratio with no apparent abnormalities. The correct introduction of the mutation was validated by PCR and sequencing (**Fig 1C**).

**Fig 2:**
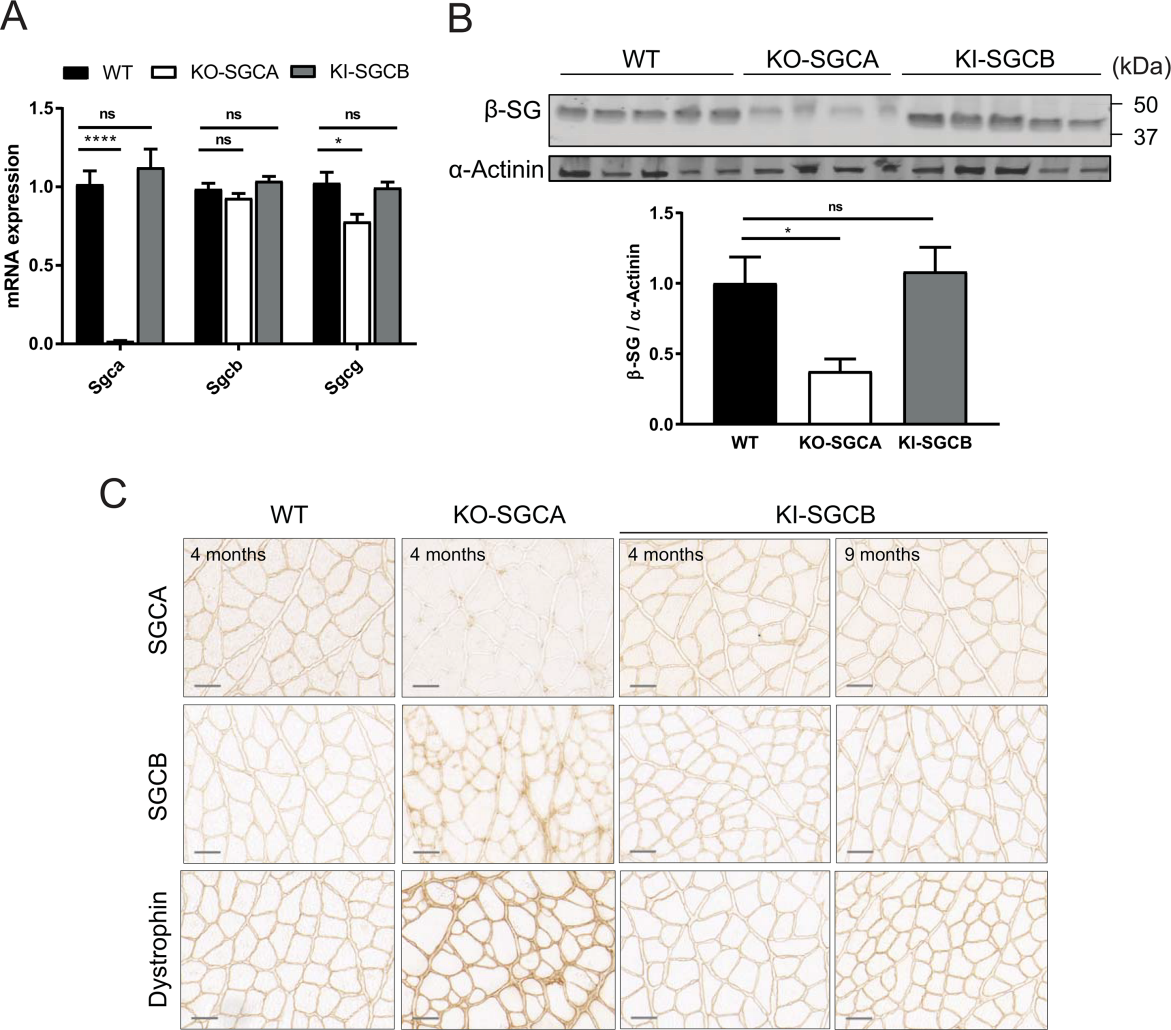
Molecular characterization of the T153R β-SG KI mouse model (n = 4 to 6 per group). **(A)** mRNA expression for α-SG, β-SG and γ-SG as defined by qPCR. No significant decrease of gene expression in the T153R β-SG KI mice was observed whereas levels of α-SG and γ-SG are substantially reduced in α-SG KO. **(B)** Protein expression of β-SG was evaluated by Western-blot performed on muscles sections form WT, α-SG KO and T153R β-SG KI mice at 4 months of age. The β-SG protein level was unaffected in T153R β-SG KI mouse, whereas it was highly decreased in α-SG KO mice. Alpha-actinin was used as loading control. **(C**) The localization of several DGC members was studied by immunostaining on muscles sections form WT, α-SG KO and T153R β-SG KI mice at 4 months of age. Immunostaining at 9 months of age is also shown for T153R β-SG KI mice. The T153R β-SG mutant protein was present at the muscle fiber membrane at 4 and 9 months and did not seem to perturb the DGC assemblage as showed by the correct membrane localization of dystrophin and α-SG. Size bar: 50μm.

The consequences of the mutation on β-SG and other members of the SG complex were determined at mRNA and protein levels by quantitative PCR (qPCR) and immunoblot analysis using the quadriceps muscle from the resulting T153R β-SG KI mice. Wild-type (WT) and α-sarcoglycan knock-out (α-SG KO) mice were used as positive and negative controls, respectively. Unlike α-SG KO mice, expression levels of the sarcoglycan genes were similar between T153R β-SG KI and WT mice, indicating that the presence of the mutation neither affects the expression and stability of the mRNA and of the mutated protein, nor of the other sarcoglycans of the complex (**Fig 2A and 2B**). The T153R β-SG protein localization, as well as of the other members of the SG complex, was then analyzed by immunostaining on muscle sections from quadriceps at 4 and 9 months of age. WT and α-SG KO mice were used as controls. The results showed that the β-SG mutant protein and other components of the DGC complex are correctly localized at the muscle membrane (**Fig 2C**), indicating that the expression of this β-SG mutant in mouse muscle do not perturb the DGC assemblage. This observation contrasts with the human situation where the biopsies showed a complete absence of the protein and of the complex at the membrane [21].

**Fig 3:**
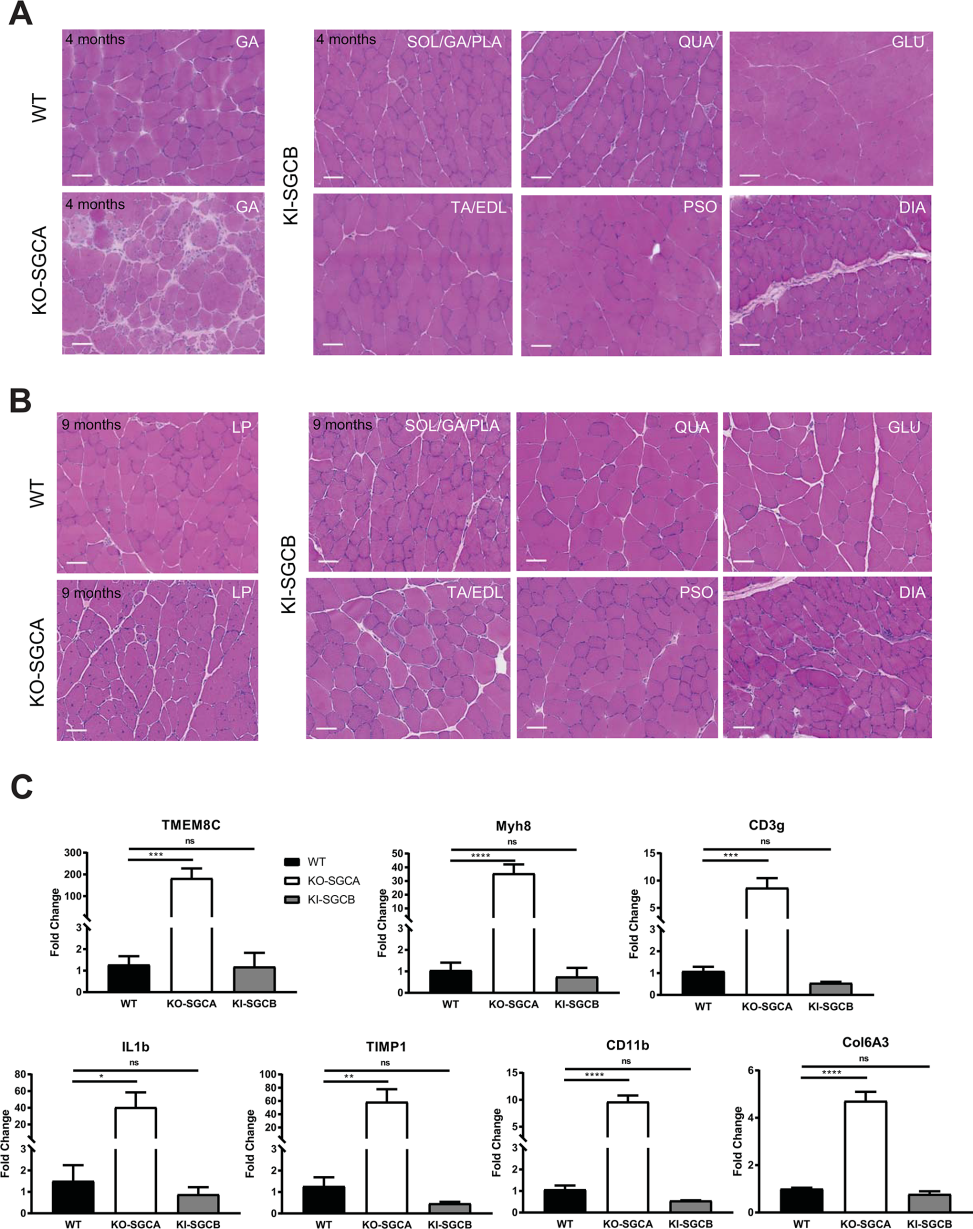
Histological characterization of the T153R β-SG KI mouse model (n = 4 to 6 per group). **(A and B)** Histological analysis of muscles from WT, α-SG KO and T153R β-SG KI mice was performed on muscle sections collected at 4 **(A)** and 9 **(B)** months of age and showed a lack of a visible muscle dystrophic features in T153R β-SG KI mice such as fibrosis and centronucleated fibers. Size bar: 50μm. **(C)** Elements of the muscular dystrophy processes were determined by RT-qPCR in WT, α-SG KO and T153R β-SG KI mouse model at 9 months of age, showing no increase in the KI model as demonstrated by the expression of markers of regeneration (TMEMC8 and Myh8), inflammation (CD3g, IL1b, TIMP1 and CD11b) and fibrosis (Col6A3).

Histological analysis of various muscles [including psoas (PSO), gastrocnemius (GA), gluteus (GLU), quadriceps (QUA), tibialis anterior + extensor digitorum longus (TA/EDL), soleus + gastrocnemius + plantaris (SOL/GA/PLA), diaphragm (DIA) and heart (HEART)] collected at 4 and 9 months of age in mice of both sexes demonstrated a lack of a visible histological dystrophic phenotype (**Fig 3A and 3B and S1A and S1B Fig**). To confirm further the absence of a dystrophic process occurring in the model, we investigated by RT-qPCR the expression level of genes known to be dysregulated and associated with the regenerative, inflammatory and fibrotic processes present during the dystrophic remodeling (**Fig 3C)**. We selected TMEM8C and Myh8 for determination of the level of regeneration [22, 23], CD3g, IL1b, TIMP-1 and CD11b for the inflammation [23–25] and Col6A3 for fibrosis [23, 26]. These markers were significantly dysregulated in the α-SG KO mice, confirming their usefulness in muscular dystrophy characterization. However, no difference was observed between WT and T153R β-SG KI mutant animals for any of these markers.

**Fig 4:**
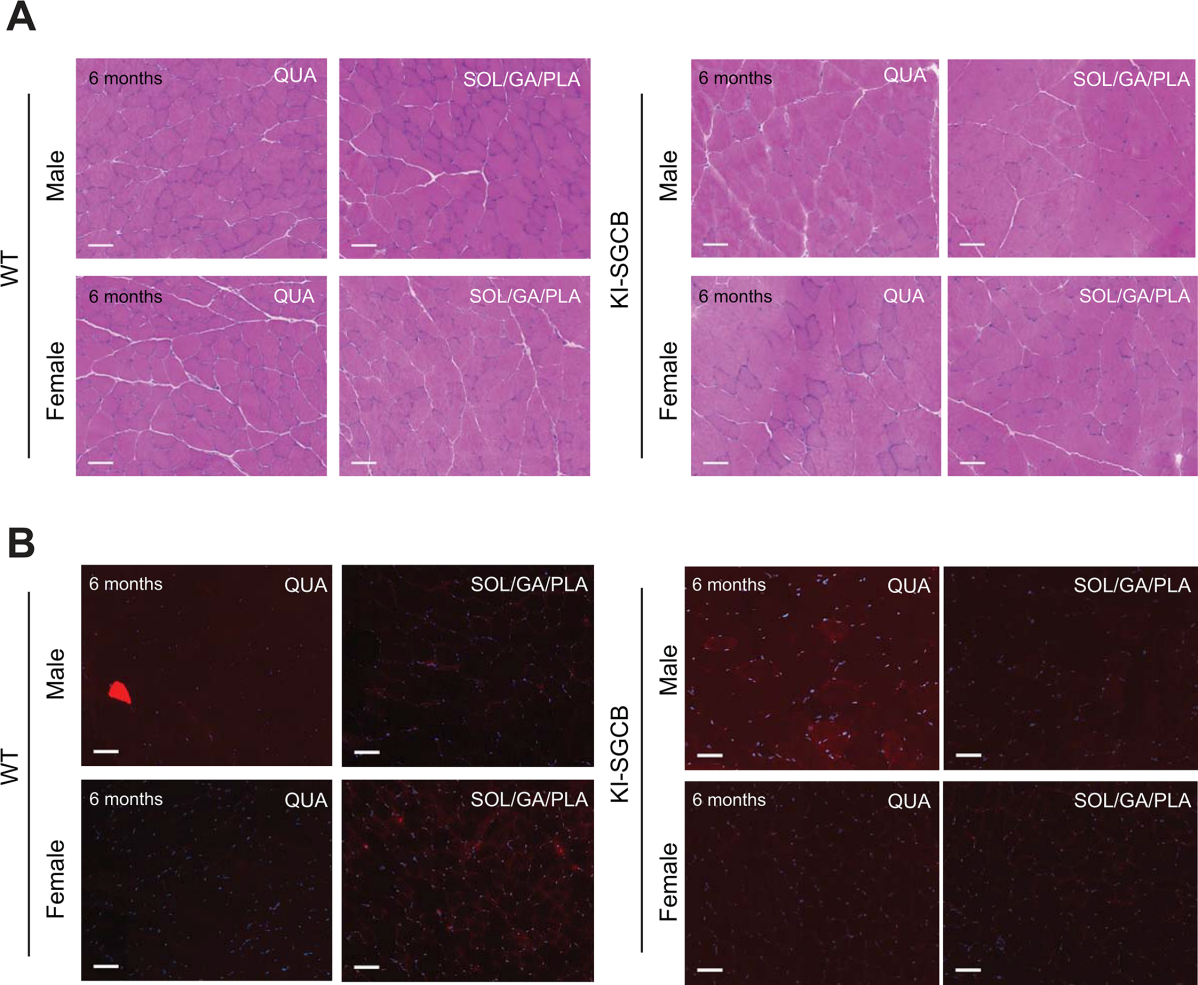
Analysis of eccentric exercise in T153R β-SG KI mice (n = 5 male and 5 female per group). **(A and B)** Analysis of muscle sections collected from male and female T153R β-SG KI mice at 6 month of age subjected to eccentric exercise showed lack of visible muscle abnormalities **(A)** and absence of visible Evans Blue positive fibers **(B)**. Size bar: 50μm.

To further define whether the introduction of the mutation induces or not a mild deficit that would appear only in challenging conditions, we performed an *in vivo* experiment in settings previously shown to be deleterious in sarcoglycan animals [12, 27]. Six-month-old male and female mice were subjected to an eccentric exercise on a treadmill three consecutive days and sacrificed 24h after the last stress. No significant difference in running was observed between WT and KI mice. Furthermore, examination of both Evans Blue positive fibers and histological aspect of the sections indicated no differences compared to wild-type, indicating that even in stressful conditions, the introduction of the mutation in the β-sarcoglycan gene has no deleterious consequences in mice (**Fig 4**).

## Discussion

All the results presented above indicate a failure in reproducing the human dystrophic phenotype observed in LGMD2E patients in the new KI mouse model. The data obtained on the expression and membrane localization of the mutated sarcoglycan were similar to the ones described previously in the α-SG H77C mouse models. In both cases, the mutation does not affect the targeting at the membrane of the corresponding protein and the assemblage of the sarcoglycan and DGC complex at the membrane, leading to the absence of phenotype in the skeletal muscle even in stressful conditions as well as in the heart, a tissue where β-SG is highly expressed [28] and that can present mild cardiac abnormalities in some β-sarcoglycanopathy patients[29, 30]. Therefore, we can conclude that, contrarily to the human R77C α-SG and T151R β-SG mutants, which are both prematurely degraded in the human context, the corresponding murine mutants fail to do so in a mouse system.

Regarding the mutation at the 77 position of α-SG, it was possible to argue that the difference in amino-acid residue at that position between mouse and human (histidine and arginine, respectively) may modify the local organization of the protein, leading to a lower structural impact of the mutation in mouse and preventing the mutant to be considered as being misfolded by the mouse ERAD pathway. In the case of β-SG, the residue is identical in both species and the homology level at the vicinity of the mutation is high with only a change 6 and 25 residues downstream of the mutation (**Fig 1A**). Unfortunately, experimental structural data is not available for β-SG and an attempt at *in silico* modeling of the mutation structural consequences proved to be unsatisfactory due to low quality of prediction. Overall, it appears difficult to explain the failure in recreating the human pathology of these mouse models solely by structural modification. Therefore, alternatively but not exclusively, one can hypothesize that the quality control system of the mouse could be less stringent than the human one. The folding and maturation of the sarcoglycans occur in the endoplasmic reticulum, the location of the ER quality control machinery. This system consists mainly of protein folding chaperones and modifying enzymes that ensure the proper folding, post-translational modifications such as glycosylation and disulfide bond formation, and finally control the quality of the folded proteins [31]. To the best of our knowledge, it seems that very few comparative studies of proteomes between the two species have been reported, especially for this specific cellular compartment. It is therefore difficult to speculate on a specific hypothesis concerning the protein quality control system that could explain our data.

The failure to reproduce a human phenotype in mice as well as in translating therapeutic efficiency from pre-clinical data obtained in mouse to clinical settings is, of course, not unprecedented, underlying the evolutionary difference in the mouse and human biology. Absence of phenotype can sometimes be seen in KO animals (i.e. in a number of metabolic diseases as reviewed by [32]), indicating either redundancy or accessory function of the corresponding protein in the species. However, in other cases as for sarcoglycans, it is clear that the protein is not dispensable when considering the important phenotype in the α-SG and β-SG KO models [16, 33]. Furthermore, we demonstrated that the mechanism of the loss of function in the case of human missense mutation is not related to the function of the protein but to its premature degradation and deficient trafficking. Such impairment of the mutated protein, albeit at a lesser extent than for sarcoglycans, was also described to be species dependent in cystic fibrosis, a prototypic misfolding disease [34]. Therefore, understanding the species differences in protein quality control pathways will give possible explanations why mouse models keep failing in recapitulate the human pathological phenotypes. In particular, a deeper knowledge about the ERQC and ERAD systems of the mouse may enable scientists to recreate more faithfully human diseases in the perspective of understanding the pathological mechanism and developing therapeutic strategies. Alternatively, models in less conventional species such as the rat, ferret, dog or pig were shown to more faithfully recreate all the human phenotypes of a disease [35] and are starting to be more widely used thanks to the change in the paradigm of their generation by the CRISPR/Cas9 revolution.

## Supporting information

S1 Fig Histological characterization of heart of the T153R β-SG KI (n = 4 to 6 per group). **(A and B)** Histological analysis of heart muscles from WT, α-SG KO and T153R β-SG KI mice **(A)** and Sirius red staining of T153R β-SG KI mice heart muscles **(B)** showed a lack of a visible heart muscle dystrophic features in T153R β-SG KI mice at 4 or 9 months of age. Size bar: 50μm.
(EPS)

S1 File ARRIVE checklist. Reporting *in vivo* experiments of the manuscript.
(DOCX)
