# Supplementary material for: Different outcome of sarcoglycan missense mutation between human and mouse
Source: PLoS One. 2018 Jan 23;13(1):e0191274. doi: 10.1371/journal.pone.0191274 (PMC5779665; doi:10.1371/journal.pone.0191274)

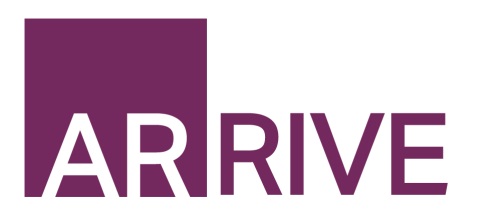


The ARRIVE Guidelines Checklist

Animal Research: Reporting In Vivo Experiments

Carol Kilkenny^1^, William J Browne^2^, Innes C Cuthill^3^, Michael Emerson^4^ and Douglas G Altman^5^

*^1^The National Centre for the Replacement, Refinement and Reduction of Animals in Research, London, UK, ^2^School of Veterinary Science, University of Bristol, Bristol, UK, ^3^School of Biological Sciences, University of Bristol, Bristol, UK, ^4^National Heart and Lung Institute, Imperial College London, UK, ^5^Centre for Statistics in Medicine, University of Oxford, Oxford, UK.*

|  | | ITEM | RECOMMENDATION | Section/ Paragraph |
| --- | --- | --- | --- | --- |
| 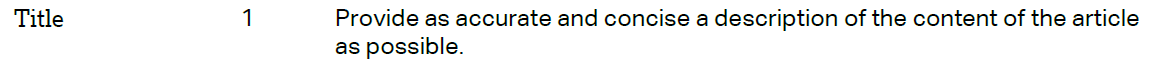 | | | Title |  |
| 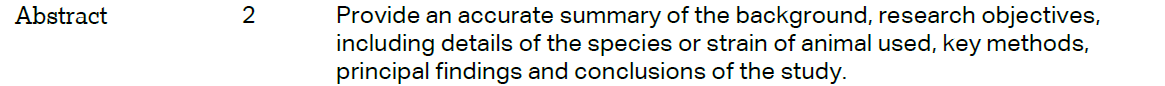 | | | Abstract |  |
| INTRODUCTION | | |  |  |
| 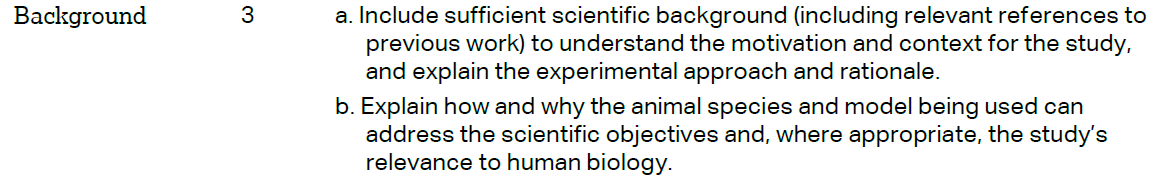 | | | Paragraphs 1-2  Paragraph 3 |  |
| 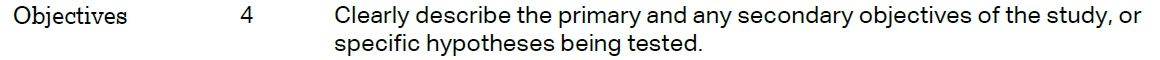 | | | Paragraph 3 |  |
| METHODS | | |  |  |
| 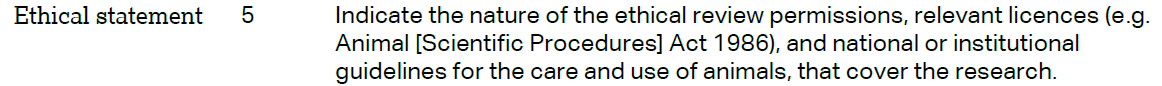 | | | Paragraph 1 |  |
| 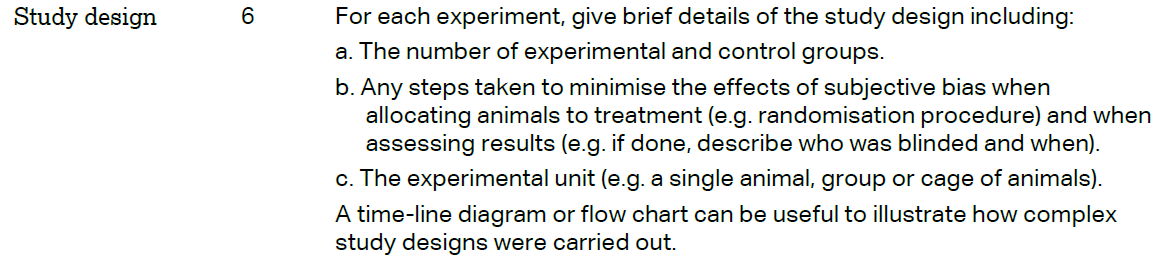 | | | Figure legends  Paragraph 5  Figure legends |  |
| 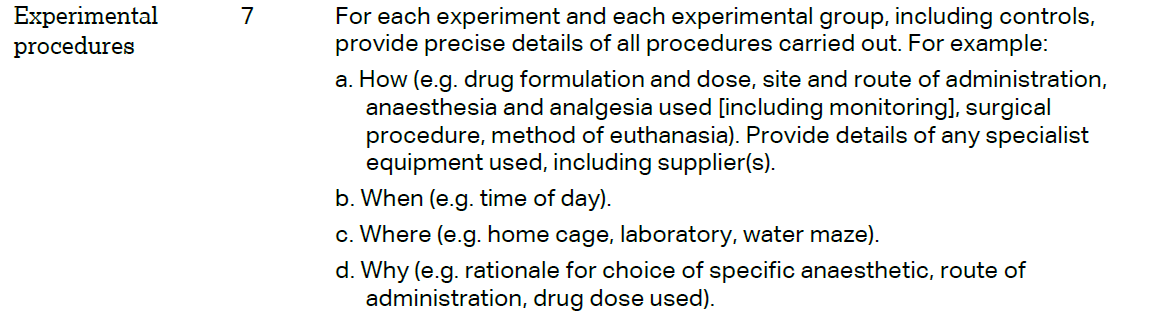 | | | Paragraphs 1 and 2 |  |
| 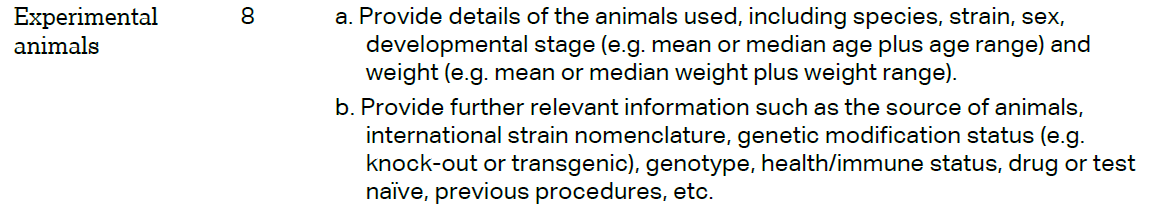 | | | Paragraphs 1 and 2 |  |

The ARRIVE guidelines. Originally published in *PLoS Biology*, June 2010^1^

| 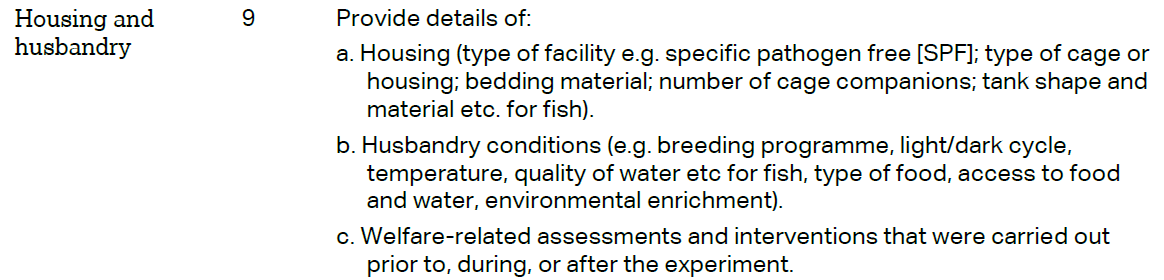 | Paragraph 1 | |
| --- | --- | --- |
| 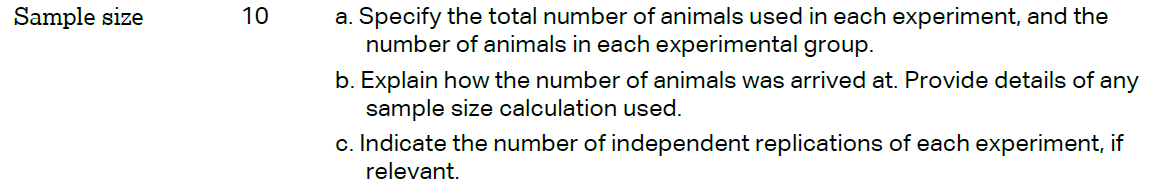 | Paragraph 2  Figure legends | |
| 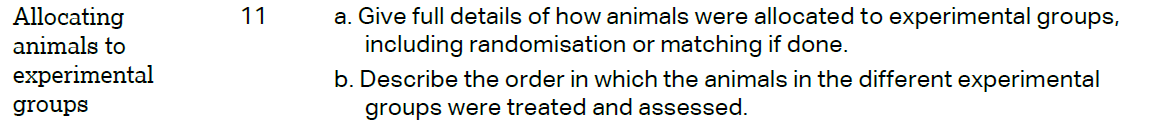 | Paragraph 2 | |
| 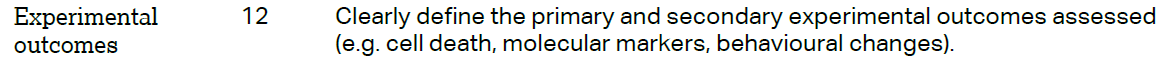 | Result paragraph 4 | |
| 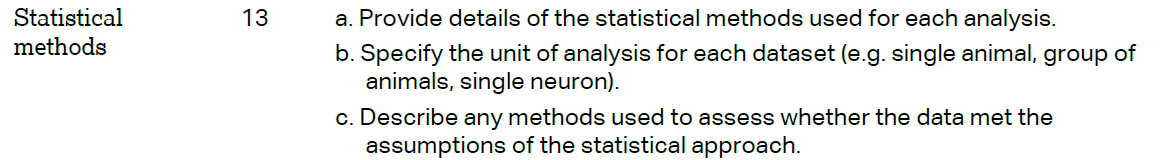 | Paragraph 7 | |
| RESULTS |  | |
| 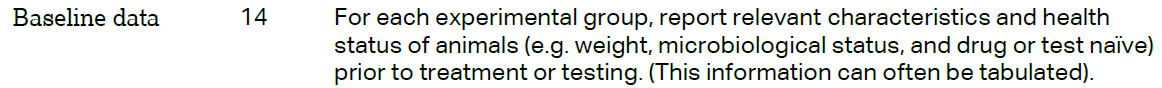 | Methods  Paragraph 1 | |
| 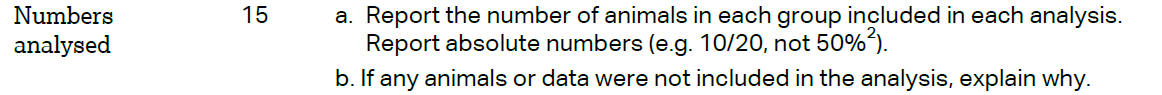 | Figure legends | |
| 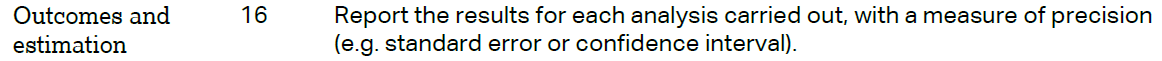 | Figures 2 and 3 | |
| 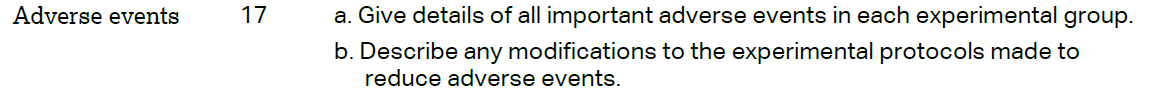 | None to report | |
| DISCUSSION |  | |
| 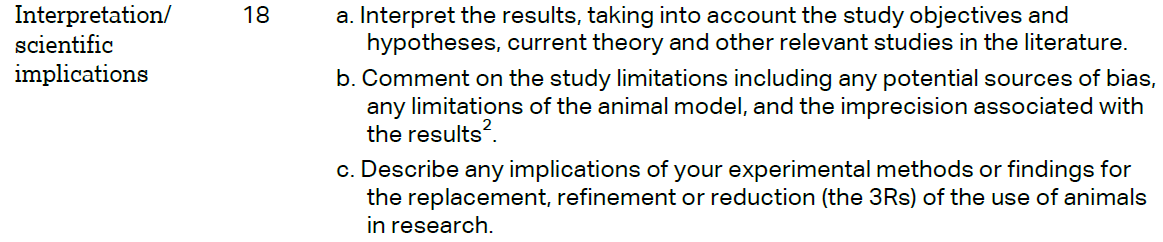 | Thoughout  Thoughout  Paragraph 3 | |
| 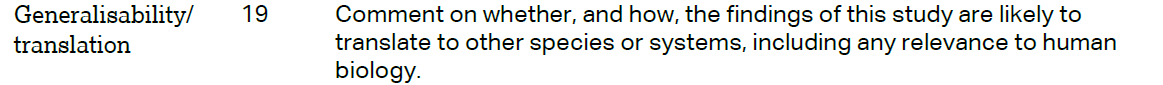 | Paragraph 3 | |
| 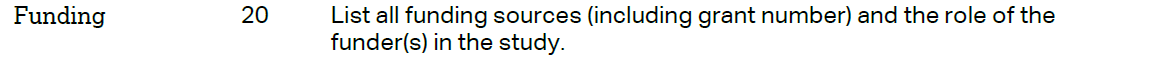 | | Funding section |


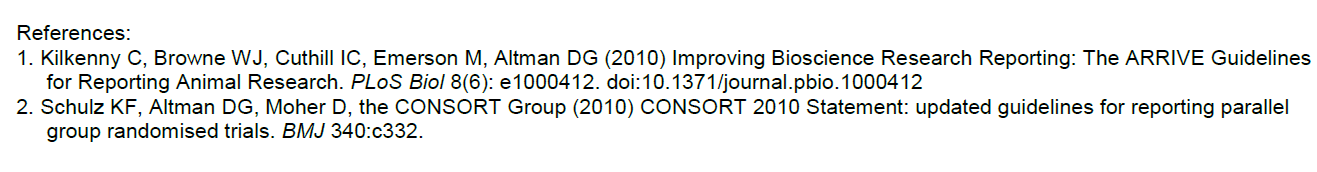

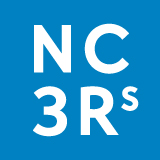

Supplement: S1 File — Reporting in vivo experiments of the manuscript. (DOCX) [file pone.0191274.s002.docx]
